# Information and Agreement in the Reputation Game Simulation

**DOI:** 10.3390/e24121768

**Published:** 2022-12-03

**Authors:** Viktoria Kainz, Céline Bœhm, Sonja Utz, Torsten Enßlin

**Affiliations:** 1 Max Planck Institute for Astrophysics, Karl-Schwarzschildstraße 1, 85748 Garching, Germany; 2 Faculty of Physics, Ludwig-Maximilians-Universität, Geschwister-Scholl Platz 1, 80539 Munich, Germany; 3 School of Physics, The University of Sydney, Physics Road, Camperdown, Sydney, NSW 2006, Australia; 4 Leibniz-Institut für Wissensmedien, 72076 Tübingen, Germany; 5 Faculty of Psychology, University of Tübingen, 72074 Tübingen, Germany; 6 Excellence Cluster Origins, Boltzmannstr. 2, 85748 Garching, Germany

**Keywords:** sociophysics, information theory, agent-based modeling, reputation dynamics, computational psychology

## Abstract

Modern communication habits are largely shaped by the extensive use of social media and other online communication platforms. The enormous amount of available data and speed with which new information arises, however, often suffices to cause misunderstandings, false conclusions, or otherwise disturbed opinion formation processes. To investigate some of these effects we use an agent-based model on gossip and reputation dynamics with 50 agents, including Bayesian knowledge updates under bounded rationality and up to the second-order theory of mind effects. Thereby, we observe the occurrence of reputation boosts from fake images, as well as the advantage of hiding one’s opinion in order to become a strong information trader. In addition, the simulations show fundamentally different mechanisms for reaching high agreement with others and becoming well-informed. Additionally, we investigate the robustness of our results with respect to different knowledge-update mechanisms and argue why it makes sense to especially emphasize the margins of distribution when judging a bounded quantity such as honesty in a reputation game simulation.

## 1. Introduction

In recent years, the fraction of most people’s time spent on the internet has grown dramatically, as the use of social media as communication and information networks has gained much importance. To be connected in the way we are is one of the outstanding characteristics of a modern lifestyle and strongly influences the dynamics of public opinions and values. As a forward striving society, it is imprinted in human nature to use all our capabilities to adapt to that modern, complex online world, both as private individuals and large economic players. Digital media provide many advantages such as access to much more information than at any time before or being able to address a huge number of people almost effortlessly. Nevertheless, these advantages also conceal risks, which are becoming increasingly apparent. There might be intentional deception in order to misguide specific discussions online, but even without any deliberately deceitful actors, the pure complexity and unmanageableness of social media can lead to undesired effects, such as filter bubbles, echo chambers, and many more.

Such effects that arise without any kind of attention steering, deliberate spreading of misinformation, or specific manipulation will be investigated in the following work. By simulating communication dynamics only based on the assumption that agents do not necessarily communicate truthfully all the time we will replicate phenomena that are known in real-life sociology. We have already done so in previous works on reputation game simulations [1], where several types of agents with different strategies were investigated. However, in this work we will only focus on one kind of agent, called ordinary agents, but use significantly larger system sizes of 50 agents. The main attention of this work thus is on emerging phenomena that arise naturally in large social groups, without any deliberately disturbing characters. For this, the reputation game simulation provides a good starting point because although the simulation becomes more complex with the number of participating agents, we are still able to track and understand every single decision the agents make. This detailed approach allows us to understand social phenomena on the basis of all the agents’ individual experiences, which in turn enables us to find causes for evolving social structures in the simulations on a microscopic, individual level. In the same way, however, in some situations it might not be the individuals who cause certain phenomena, but rather the whole social structure itself, which is why we need the larger setup of 50 agents to investigate this as well. So far, most related works on reputation dynamics only take care of either side. For example, often simplistic update algorithms based on DeGroot learning [2] are used in order to model the opinion formation process in the agents’ minds [3,4,5,6]. This, however, neglects the effects of uncertainty both about the information source and the transmitted content and does not consider the need for a theory of mind at all. Another common method is acceptance thresholds, where agents either trust or distrust the information source completely depending on whether or not the communicated information seems to be credible [7]. Although such models use highly over-simplified mental models of each individual agent, they are able to perform simulations with large system sizes, typically on the order of a few thousand agents. On the other hand, there are also works with more advanced mental models based on Bayesian logic and also including a theory of mind [8,9,10,11,12]. They, however, typically only deal with small system sizes of a few individuals and focus on those individuals’ perceptions and decisions. Only the authors of [12] perform simulations with 50 agents, but again without a theory of mind. Additionally, in order to handle the complexity of probabilistic mental models, all states are typically discrete, which limits the number of possible world states and reduces computational costs [8,9,10,11,12]. In comparison to these models, the reputation game aims to fill this gap between advanced mental models for each individual agent and still capture large system sizes. Therefore, we combine approximate Bayesian updates (fully Bayesian updates would not even be realistic, since humans are not perfect Bayesian reasoners. On the contrary, humans are known to use mental shortcuts in order to deal with the high complexity and incalculability of every-day-life [13]), a DeGroot based theory of mind, and continuous world states with a comparatively large system size of 50 agents. With this setup, the reputation game is able to both model each agent’s psychology on a detailed level and to study large-scale sociological phenomena.

## 2. The Reputation Game

The reputation game is an agent-based socio-psychological simulation that captures gossip dynamics about the agents’ reputations. In the following work, we have a predefined set of agents that communicate with each other either in one-to-one or in one-to-many conversations. We assume that all the agents are of the same kind, called ordinary agents since they do not have any specific strategy for behaving advantageously and most choices are done randomly. The only differences between the agents are their personal character traits, where the most important one is an agent’s intrinsic honesty, i.e., the frequency with which it lies. On a second level, the agents’ intrinsic honesties are also the topics of all conversations, meaning that the agents are constantly gossiping about their and others’ honesty while trying to find out the truth. In order to implement a simplified, but working example of such communication networks we base our simulation on a few assumptions. One is the presence of **deception**, which does not have to follow a specific malicious plan in the first place, but naturally arises in human communication [14,15], e.g., due to personal interests in the case of the reputation game. Next, as soon as humans interact with each other we automatically consider others’ current mental states, as well as certain environmental circumstances in order to interpret the communicated words correctly. Since also the agents in the reputation game constantly communicate and judge each other’s credibility, we introduce a rudimentary **theory of mind**, which allows the agents to keep track of others’ belief states. Additionally, for the above environmental circumstances, we allow the agents to adapt their current perception of the **social atmosphere** which enables them to interpret messages in different ways depending on the current status of their surroundings. Although humans are extremely good at capturing various signs and details during a conversation, we do not remember all this information for a long time. Instead, we use mental shortcuts to efficiently filter out important information and forget or neglect irrelevant parts [13]. Analogously, we equip the agents with an **approximate Bayesian reasoning** that allows an optimal update with all available information in the first place, but afterwards forces the agents to keep only a compressed version of their obtained knowledge in their memory by focusing on the most important aspects. By performing such conservations and updates hundreds of times during a simulation, the agents learn about others’ honesties piece by piece, as precisely as possible in this lie-distorted system. In the following, this process is described in a more rigorous fashion, as well as the agents’ decision-making processes, i.e., how to lead a conversation and how to deal with a received message.

### 2.1. Setup

At the beginning of each simulation, there is a set A of n=50 agents that have three personality traits, which characterize the individual agents: their honesty *x*, their shyness *S*, and their friendship affinity *F*. An agent does not know any of these quantities at the beginning, but tries to find out its own and the others’ honesties throughout the game. The rest of the simulation is organized in rounds, where in each round every agent initializes one conversation. Thereby the agents choose randomly whether to speak to only one other agent or to communicate a message to several agents at once. In a one-to-one conversation the addressed agent will subsequently also answer on the same topic, whereas one-to-many communications are supposed to be only one-way, similar to broadcasting messages on social media platforms. After that, they decide on the topic of conversation as well as the message’s content, which will finally be communicated. Before we explain all those steps and the involved decision-making processes in more detail, we want to emphasize a few key concepts on which the reputation game is based on.

### 2.2. Key Concepts

As already mentioned, agents have intrinsic personality traits that characterize every single agent and make them behave differently: honesty, shyness, and friendship affinity. In the reputation game, **honesty** is the most important quantity because it also serves as a conversation topic and is the quantity the agents actively think about and store information on. The honesty *x* of an agent defines the frequency with which an agent makes honest statements or lies, respectively. It ranges from x=0, meaning that every single communication of an agent is a lie, and x=1, meaning that the agent is honest every time. During a simulation the agents try to identify when others have been honest or dishonest, i.e a network of mutual honesty assignments arises, which we associate with **reputation**. The reputation of an agent is thus defined as the (average) honesty one or several other agents assign to it and is the key quantity the agents want to optimize for themselves. Besides their intrinsic honesty, every agent has its personal **shyness**, and **friendship affinity** value. Both influence their interactions with others and help to orient the agent in a large social group. A high shyness value for example makes an agent concentrate on agents it knows well, i.e., is well acquainted with. **Acquaintance** in the reputation game is, therefore, measured by the number of conversations two agents had with each other, as well as the number of messages an agent got about another one. Both for choosing a conversation partner and a topic, the agents are influenced in their decision by an acquaintance. The former accounts for the intuitive understanding of shyness in the sense of having more or less hesitation to talk to strangers. The latter ensures that when choosing a topic of conversation, the agents do not talk about someone they have no information on, because this would otherwise lead to many useless conversations where no information is actually transmitted. Friendship affinity is based on a similar principle, but influences only the choice of the conversation partner and not the topic. A **friendship** of an agent *i* to another one *j* in the reputation game is a measure for how many positive statements agent *i* heard agent *j* saying about itself compared to what others say on average. It will, therefore, later on also determine whether to lie positively or negatively about someone, as an agent wants to promote its friends such that they are believed when expressing their positive view about that agent and demote its enemies accordingly. Friendship affinity also makes the agents preferentially choose their friends when deciding who to speak to, which simulates the desire for self-affirmation that is also present in human behavior [16,17]. How strong this effect is, however, is different for every agent and imprinted in their personal friendship affinity value.

Moreover, agents are also information processing entities and as such, they need specific concepts and structures to think of. In the reputation game simulation there are three such concepts: credibility, theory of mind, and the social climate. **Credibility** is the most intuitive one, as agents are constantly confronted with potential lies and need to judge the credibility of every single piece of information they get. The credibility of a message is, therefore, the probability of that message resulting from an honest communication given all data that are additionally available to the agent. For example these include the agent’s knowledge of the honesty of the speaker, other knowledge about the agent’s or speaker’s worldview, or the message’s content itself. In order to asses others’ worldviews at all, the agents possess a rudimentary **theory of mind**, which they update during the conversations and is another key feature in the reputation game. For example, the agents need to estimate others’ opinions in order to appropriately target their lies or to distinguish between others’ making an honest statement and pursuing a manipulating strategy. Last but not least, the agents have to adapt to a constantly changing environment, since not only their own, but all the agents’ states permanently change and with it the behavior of the whole group. To recognize this change in behavior we introduce the concept of surprise. The surprise an agent experiences when receiving a message is defined as the Kullback–Leibler divergence [18] between the agent’s prior belief state and the content of the incoming message. It is, on the one hand, used to simulate skepticism in the case of a too extreme message, but on the other hand, its scale needs to be adaptive since the threshold between a typical surprise of an honest or dishonest statement is not a priori clear and needs to be learned. This way very different regimes of **social climates** appear, ranging from a frozen one, where the opinions of all agents are quite static and also lies can not be far from the (commonly believed) truth in order to go undetected, to a toxic social climate, where opinions might change very chaotically and both honest and dishonest statements undergo huge dispersion.

### 2.3. Messages and Information Representation

The communications between agents are the core of the simulation. To understand how agents communicate in the first place, we start by looking at their internal information representation. In order to judge someone’s honesty, the agents keep track of two parameters μ and λ, which can be seen as the assumed number of honest and dishonest statements an agent seems to have made, respectively. As the beta distribution arises naturally when dealing with binary data (such as honest or dishonest statements) and is also used by many other agent-based models on reputation dynamics, e.g., [19], the agents assume the probability distribution based on their knowledge I=(μ,λ) to be

(1) $$\begin{array}{c} P\left(x|I\right)=\mathrm{Beta}\left(x|\mu ,\lambda \right)=\frac{x^{\mu }{\left(1-x\right)}^{\lambda }}{B(\mu +1,\lambda +1)} \end{array}$$

about someone’s honesty *x*. So an agent *i* stores information $I_{ij}=\left(\mu_{ij},\lambda_{ij}\right)$ about any other agent j∈A, where the agents assume the individual probability distributions to be independent:

(2) $$\begin{array}{c} P\left(x_{1},\ldots ,x_{n}|I_{i}\right)=\prod_{j\in A}P\left(x_{j}|I_{ij}\right). \end{array}$$

When transmitting a message, the same form of information as for their internal belief representation is used. A message $J_{a\overset{c}{\to }b}$ from the speaker *a* to its interlocutor *b* about a topic *c* is thus determined by the same two parameters $J_{a\overset{c}{\to }b}=\left(\mu_{a\overset{c}{\to }b},\lambda_{a\overset{c}{\to }b}\right)$. Thereby a whole probability distribution is transmitted, always including uncertainty about the conversation topic’s honesty. How exactly a message $J_{a\overset{c}{\to }b}$ is constructed in both the honest and dishonest case will be discussed in Section 2.4.

### 2.4. Communication Strategy

Communicating in the reputation game includes three major decisions: who to talk to, who’s honesty to talk about, and what to say.

#### 2.4.1. Choosinga Conversation Partner

First, the conversation initiating agent *a* decides on a conversation partner, which can be either a single other agent, called b∈A\{a} or a subset of agents $\left(b_{1},b_{2},\ldots \right)\subset A\backslash \left\{a\right\}$. The probability of agent *a* to choose any other agent *b* as conversation partner is given by

(3) $$\begin{array}{c} P\left(a\overset{\cdot }{⇆}b|a\overset{\cdot }{⇆}\cdot \right)\propto \left(1-\delta_{ab}\right)r_{ab}^{S_{a}}f_{ab}^{F_{a}}, \end{array}$$

where $r_{ab}=r_{ab}^{m}+Qr_{ab}^{c}$ is agent *a*’s acquaintance with agent *b*, consisting of the number of messages agent *a* has heard about *b*, $r_{ab}^{m}$, and the number of conversations they had, $r_{ab}^{c}$, weighted by a factor of Q=10 to which conversations are considered more important. This is an assumption based on a trade-off between two regimes. On the one hand, *Q* needs to be >1 to ensure that agents preferentially choose others they have already talked to. Having heard of someone should only give a slight chance of getting to know new agents, similar to getting to know friends’ friends in reality, but the acquaintance to first-order friends should still be higher. On the other hand, *Q* must not be extremely large, because then only small groups would form at the beginning and remain that way later on. Q=10, therefore, is a temporarily chosen assumption that yields reasonable, middle-sized groups. $f_{ab}=\frac{\pi_{ab}+1}{\pi_{ab}+\nu_{ab}+2}$ is the friendship agent *a* has to *b*, measured by the number of comparably positive and negative statements *a* has heard *b* saying about itself, $\pi_{ab}$ and $\nu_{ab}$, respectively, and applying Laplace’s rule of succession to estimate the rate of friendly/unfriendly statements expected. Therefore, the agents preferentially choose someone they already know (how important that is for the individual agent is measured by its shyness $S_{a}$) and someone who is positively minded towards them (again with different importance to the individual agents measured by their friendship affinity $F_{a}$). $\delta_{ab}$ is the Kronecker delta and ensures that agents do not talk to themselves.

When not a single agent is chosen as the receiver, but a set of agents, it first has to be determined to how many agents the message will be broadcasted. This depends on the initiator’s shyness $S_{a}$, since less shy agents are considered to talk more easily to larger groups than shy agents. The number of recipients is accordingly drawn from the following distribution:

(4) $$\begin{array}{c} P\left(\mathrm{Number}\;\mathrm{of}\;\mathrm{recipients}=N_{b}\right)=\frac{N_{b}^{-S_{a}}}{\sum_{N_{b}=2}^{n-1}N_{b}^{-S_{a}}}. \end{array}$$

One could argue that including the possibility $N_{b}=1$ here as well and performing a two-way communication (including an answer) if only one conversation partner is chosen might be more consistent. However, in this case extroverted agents, e.g., ones with a low shyness value, would only rarely get answers from their conversation partners, which would be important to obtain information, though. We, therefore, choose the fraction of one-way and two-way communications to be independent of the agent’s shyness value, such that all agents have the same chance to collect information in the first place. Once the number of recipients $N_{b}$ is defined, the initiator determines the probability of choosing every single agent except for itself as conversation partner according to Equation (3) and draws $N_{b}$ samples from the resulting distribution.

#### 2.4.2. Choosing a Conversation Topic

Second, the conversation initiator has to choose a topic, which is the honesty of any other agent c∈A, including *a* itself. As mentioned in Section 2.2, the topic choice only depends on the agents’ acquaintance, such that at least the conversation initiator has actually had any contact with the topic and there exists some non-trivial opinion to transmit. Therefore, the probability of an initiator *a* choosing a conversation topic *c* is given by

(5) $$\begin{array}{c} P\left(a\overset{c}{⇆}b|a\overset{\cdot }{⇆}b\right)\propto r_{ab}^{S_{a}}. \end{array}$$

#### 2.4.3. Designing a Message

In the following, we now talk about the speaker in general instead of the conversation initiator like before. This is convenient for both the primary message and the subsequent response message, because in the latter, the initial receiver becomes the speaker and vice versa. All following equations will name the speaker *a* and the receiver *b*, but they apply in the same way to the answer with speaker *b* and receiver *a*. The third part of a conversation is, to decide what to say. Here we have to distinguish between honest and dishonest statements. In case the speaker is honest (which happens with the frequency $x_{a}$) it will simply transmit its current true belief on the topic *c*, i.e., $J_{a\overset{c}{\to }b}=I_{ab}$. In case the speaker is dishonest (which happens with frequency $\left(1-x_{a}\right)$), the construction of a message is more complicated, because in principle, there are infinitely many options on how to lie. The lying strategy in the reputation game is based on the assumption that messages should not be freely invented, but increase their own reputation. Key components to achieve this goal are, on the one hand, not to be caught lying, because this would harm the own reputation, and on the other hand, to manipulate the receiver’s opinion in the desired direction. The former is achieved by making the dishonest message similar to the receiver’s current opinion, because then it will sound familiar and believable. If there is only one receiver, the speaker can use its theory of mind to estimate the momentary opinion of *b* about *c*, $I_{abc}=\left(\mu_{abc},\lambda_{abc}\right)$ and only shift it slightly. If there are several receivers at once, the speaker has to build an average of all the receivers’ belief states. In order to be believed by as many as possible the speaker, therefore, should approximately match its statement with this socially accepted group opinion. However, not every recipient might be of equal interest to the speaker, which is why the average opinion is a weighted superposition of all the receivers’ individual opinions. The weights are hereby the same as for choosing the corresponding agent as a conversation partner given by Equation (3). The weighted superposition of all receivers’ opinions

(6) $$\begin{array}{c} \begin{array}{cc} P(x|I_{ab_{1}c},I_{ab_{2}c},\ldots ) & =\sum_{i=1}^{N_{b}}w_{i}\frac{x^{\mu_{aic}}{\left(1-x\right)}^{\lambda_{aic}}}{B(\mu_{aic}+1,\lambda_{aic}+1)}\;\;\;\;\mathrm{with}\\ w_{i} & \propto r_{ai}^{S_{a}}f_{ai}^{F_{a}} \end{array} \end{array}$$

is then compressed into the form of a single beta function $\mathrm{Beta}(\mu_{a(b_{1},b_{2},\ldots )c},\lambda_{a(b_{1},b_{2},\ldots )c})$ (for the compression there exist two similar, but not identical mechanisms that will be discussed later in Section 2.5.3 and Section 2.5.4). The resulting average opinion $I_{a(b_{1},b_{2},\ldots )c}=\left(\mu_{a(b_{1},b_{2},\ldots )c},\lambda_{a(b_{1},b_{2},\ldots )c}\right)$ is then used as a starting point for the further design of the lie in the same way as $I_{abc}=\left(\mu_{abc},\lambda_{abc}\right)$ for only one receiver. For simplicity we will only use the single-receiver case for all further calculations, having in mind that everything works the same for several receivers just by replacing $I_{abc}$ with $I_{a(b_{1},b_{2},\ldots )c}$.

Starting from $I_{abc}$, the speaker can now either shift the receiver’s opinion into a more positive or a more negative direction. Which one is used depends on what is advantageous for the speaker’s reputation. In case the speaker is sure that the topic spreads a positive image of the receiver, the latter’s reputation should be enlarged, such that its opinion gets more weight. In other words, if the speaker regards the topic a friend, i.e., $f_{ac}>\frac{1}{2}$, it should lie positively about the latter. Similarly, if the speaker regards the topic an enemy, i.e., $f_{ac}<\frac{1}{2}$ the lie should be biased negatively. Both for friends and for enemies the size of the lie depends on the strength of the friendship and enmity, respectively, i.e., the agents risk larger lies if it is about manipulating good friends’ or extreme enemies’ reputation, and risk less in case of less strong friends or enemies. About themselves, agents always make fully positive statements (pretending $f_{aa}=1$) in order to promote their own reputation as strongly as possible. When the speaker does not have any friendship or enmity data regarding the topic, i.e., $f_{ac}=1/2$ it does not disturb the message at all and transmits a white lie. In total, the transmitted message $J_{a\overset{c}{\to }b}$ in the case of a lie is

(7) $$\begin{array}{c} J_{a\overset{c}{\to }b}=\left\{\begin{array}{ccc} I_{abc}+(|2f_{ac}-1\left|\Delta \mu ,0\right) & =(\mu_{abc}+|2f_{ac}-1|\Delta \mu ,\lambda_{abc}), & f_{ac}>\frac{1}{2}\;\mathrm{or}\;a=c\\ I_{abc} & =(\mu_{abc},\lambda_{abc}), & f_{ac}=\frac{1}{2}\\ I_{abc}+(0,|2f_{ac}-1\left|\Delta \lambda \right) & =(\mu_{abc},\lambda_{abc}+|2f_{ac}-1|\Delta \lambda ), & f_{ac}<\frac{1}{2} \end{array}\right. \end{array}$$

The exact size of manipulation, i.e., the value of Δμ or Δλ, is determined by a trade-off between being small enough to not stay believable and large enough to have a significant impact. Therefore, the current social climate is the decisive parameter as it defines a social standard of how large lies typically seem to be in the current situation. In a frozen climate, for example, both Δμ and Δλ would be rather small, whereas, in a toxic social climate full of chaos, both can increase by orders of magnitude. More details on the lie construction, and especially on the determination of Δμ and Δλ, can be found in [1].

### 2.5. Receiver Strategy

Besides communicating, i.e., sending a message the second, equally important action of the agent is to receive a message, i.e., to listen and interpret it. This can be separated into two major steps: judging the message’s credibility and accordingly updating their own knowledge afterward.

#### 2.5.1. Judging a Message’s Credibility

Judging the credibility of a received message means estimating the probability of that message having been honest under consideration of all available data *d*. These are the current belief state of the receiver $I_{b}$, the content of the message $J_{a\overset{c}{\to }b}$ as well as any additional signs, for example whether or not the speaker blushed or made a confession. The credibility of a message, $y_{J}$, i.e., the probability that the message was honest (h) can thus be calculated using Bayes’ Theorem:

(8) $$\begin{array}{c} \begin{array}{cc} y_{J}=P\left(h|d\right) & =\frac{P(d|h)P(h)}{P(d)}\\ & =\frac{P\left(d|h\right){\bar{x}}_{ba}}{{\bar{x}}_{ba}P\left(d|h\right)+\left(1-{\bar{x}}_{ba}\right)P\left(d|\neg h\right)}\\ & ={\left[1+R\left(d\right)\left({\bar{x}}_{ba}^{-1}-1\right)\right]}^{-1}\\ \mathrm{with}\;\;\;R(d) & =\frac{P(d|h)}{P(d|\neg h)}. \end{array} \end{array}$$

${\bar{x}}_{ba}$ hereby is the expected honesty the receiver *b* assigns to the speaker *a*, ${\langle x_{a}\rangle }_{\left(x_{a}|I_{ba}\right)}$. The likelihood ratio R(d) indicates the probability that this message has been sent to either an honest or dishonest speaker. For the credibility estimation, the agents use several criteria. First of all, they use a general bias, which is the momentary reputation of the speaker in the eyes of the receiver. If the receiver does not trust the speaker in the first place, the former is very likely to regard the message as a lie. Second, the agents use telltale signs that could reveal a lie. In the reputation game there exist two of them: blushing, as already mentioned before and confessing. Blushing occurs randomly on average with every 10th lie and is a certain indicator that the speaker was dishonest. Contrarily, confessions are a clear signal to the receiver that the speaker was honest. A confession is considered a message about the speaker itself, which revealed a more negative impression of the speaker compared to what the receiver originally believed. This is the case because if the speaker had lied, it would have tried to influence the receiver’s opinion upwards, i.e., told something better than what the receiver believed so far. The third criterion the agents use to estimate the truthfulness of the message is surprise. As already explained in Section 2.2, agents become skeptical if the received message deviates too much from their prior belief. For further details on how to calculate the likelihood ratio R(d) from these different criteria, see [1].

#### 2.5.2. Knowledge Update

Once the agents have determined the credibility of a received message $y_{J}$ they can update their own belief accordingly. Since most of the time the receivers can not be sure whether or not the message was a lie, but rather have the probability $y_{J}$ that the message was honest, they also have to partially update their knowledge for both cases. Furthermore, we have to consider that agents do not only get information about the topic of conversation, but also about the speaker. First of all, consider what the receiver can learn about the speaker. Depending on $y_{J}$, the receiver should account for an honest or dishonest statement from the speaker, as well as the possibility of getting some information on the latter’s own belief state. When we denote the receiver’s knowledge state after the update with $I_{b}^{\prime }$ we can write its posterior belief on the speaker *a* as

(9) $$\begin{array}{c} \begin{array}{cc} P(x_{a}|I_{ba}^{\prime }) & =y_{J}P\left(x_{a}|\left(\mu_{ba}+1,\lambda_{ba}\right)\right)+\left(1-y_{J}\right)P\left(x_{a}|\left(\mu_{ba},\lambda_{ba}+1\right)\right)\\ & =y_{J}\mathrm{Beta}\left(x_{a}|\mu_{ba}+1,\lambda_{ba}\right)+\left(1-y_{J}\right)\mathrm{Beta}\left(x_{a}|\mu_{ba},\lambda_{ba}+1\right). \end{array} \end{array}$$

Additionally, the agents update their theory of mind, i.e., their assumption of the others’ beliefs, by blending the newly received information into their prior knowledge. In the case that the speaker was honest, the receiver can learn something about the speaker’s true belief on the topic $I_{bac}$:

(10) $$\begin{array}{c} I_{bac}^{\prime }=y_{J}J_{a\overset{c}{\to }b}+\left(1-y_{c}\right)I_{bac}. \end{array}$$

Now, consider what the receiver can learn about the topic of conversation. Again we have to consider both cases in which the speaker has either been honest or dishonest. In the first case, the message should be believed and incorporated into the own knowledge state, whereas in the second case the message’s content should rather be ignored. This leads to the posterior probability distribution of agent *c*’s honesty as seen from agent *b*

(11) $$\begin{array}{c} \begin{array}{cc} P(x_{c}|I_{bc}^{\prime }) & =y_{J}P\left(x_{c}|\Delta J^{+},I_{bc}\right)+\left(1-y_{J}\right)P\left(x_{c}|I_{bc}\right)\\ & =y_{J}\mathrm{Beta}\left(x_{c}|\mu_{bc}+\Delta \mu^{+},\lambda_{bc}+\Delta \lambda^{+}\right)+\left(1-y_{J}\right)\mathrm{Beta}\left(x_{c}|\mu_{bc},\lambda_{bc}\right). \end{array} \end{array}$$

Here, $\Delta J^{+}=\left(\Delta \mu^{+},\Delta \lambda^{+}\right)$ is the amount of new information that seems to be in the message compared to the speaker’s last statement on the topic. This prevents the agents from taking the same opinion they hear more than once as completely new each time, but filtering out already known information. How $\Delta J^{+}$ is calculated in detail can again be found in [1].

Except for the DeGroot-like update mechanism [2] in the theory of mind (Equation (10)), the agents so far use pure Bayesian logic in order to draw as sophisticated conclusions from the available data as possible. However, this would rapidly become extremely complex due to two reasons. On the one hand, the joint updated distribution on both conversation topic and speaker, $P(x_{a},x_{b}|I_{bc}^{\prime })$, is not a product of the two marginal distributions anymore, because the communicated message makes, for example, the update on the topic dependent on the conclusions about the speaker. On the other hand, even the forms of the marginal distributions (if assumed to be independent) are not in the form of beta functions anymore, but superpositions as one can see in Equations (9) and (11). Since humans also use mental shortcuts to deal with the enormous amount of information they are confronted with in everyday life [13], we implement a limited cognitive capacity for the agents as well. Therefore, after each knowledge update where the posterior probability distributions end up being superpositions of beta functions, we force the agents to compress their new knowledge again in the form of a single beta function. This way the agents filter out which information is important to keep and which is not, and thereby concentrate on the most valuable information. For the exact implementation, however, there are different approaches of compression, of which we compare two here, namely the conservation of as much information as possible and the conservation of the supposedly most important information, reputation.

#### 2.5.3. Conservation of Most Information

In order to conserve as much information as possible while compressing a complex probability distribution $P(x|I^{\prime })$ (e.g., Equations (9) and (11) into a simpler form $P\left(x|I^{\prime \prime }\right)=\mathrm{Beta}\left(x|\mu^{\prime \prime },\lambda^{\prime \prime }\right)$ information theory suggests to minimize the Kullback–Leibner divergence (KL) $D_{\mathrm{KL}}(P\left(x|I^{\prime }\right)||P\left(x|I^{\prime \prime }\right))$ [18]. Intuitively, conserving the largest amount of information possible is a reasonable approach, but it turns out that doing so leads to the conservation of two moments, which might not be as intuitive. Minimization of the KL with respect to the parameters of the simpler probability distribution $I^{\prime \prime }=\left(\mu^{\prime \prime },\lambda^{\prime \prime }\right)$ yields

(12) $$\begin{array}{c} \begin{array}{cc} \frac{\partial \mathrm{KL}}{\partial \mu^{\prime \prime }} & =\partial \mu^{\prime \prime }\int dxP\left(x|I^{\prime }\right)\ln\left(\frac{P(x|I^{\prime })}{P(x|I^{\prime \prime })}\right)\\ & =\int P\left(x|I^{\prime }\right)P\left(x|I^{\prime \prime }\right)\cdot \partial \mu^{\prime \prime }\left(\frac{B(\mu^{\prime \prime }+1,\lambda^{\prime \prime }+1)}{x^{\mu^{\prime \prime }}{\left(1-x\right)}^{\lambda^{\prime \prime }}}\right)\\ & =\left(\psi \left(\mu^{\prime \prime }+1\right)-\psi \left(\mu^{\prime \prime }+\lambda^{\prime \prime }+2\right)\right)-{\langle \ln\left(x\right)\rangle }_{I^{\prime }}\overset{!}{=}0 \end{array} \end{array}$$

(13) $$\begin{array}{c} \begin{array}{cc} \Rightarrow & {\langle \ln\left(x\right)\rangle }_{I^{\prime }}=\psi \left(\mu^{\prime \prime }+1\right)-\psi \left(\mu^{\prime \prime }+\lambda^{\prime \prime }+2\right)\\ & {\langle \ln\left(1-x\right)\rangle }_{I^{\prime }}=\psi \left(\lambda^{\prime \prime }+1\right)-\psi \left(\mu^{\prime \prime }+\lambda^{\prime \prime }+2\right). \end{array} \end{array}$$

The last line follows from analogous minimization with respect to $\lambda^{\prime \prime }$ and ψ(x) is the digamma function. These are the expectation values of ln(x) and ln(1−x) given the more complex, superpositional probability distribution $P(x|I^{\prime })$. We can now compare those to the same expectation values, but given the simplified distribution $P(x|I^{\prime \prime })$:

(14) $$\begin{array}{c} \begin{array}{cc} {\langle \ln\left(x\right)\rangle }_{I^{\prime \prime }} & =\int dx\ln\left(x\right)P\left(x|I^{\prime \prime }\right)\\ & =\int dx\ln\left(x\right)\frac{x^{\mu^{\prime \prime }}{\left(1-x\right)}^{\lambda^{\prime \prime }}}{B(\mu^{\prime \prime }+1,\lambda^{\prime \prime }+1)}\\ & =\psi \left(\mu^{\prime \prime }+\lambda^{\prime \prime }+2\right)-\psi \left(\mu^{\prime \prime }+1\right)\;\mathrm{and}\;\mathrm{similarly}\\ {\langle \ln\left(1-x\right)\rangle }_{I^{\prime \prime }} & =\psi \left(\mu^{\prime \prime }+\lambda^{\prime \prime }+2\right)-\psi \left(\lambda^{\prime \prime }+1\right) \end{array} \end{array}$$

We conclude that for both probability distributions the expectation values are the same, i.e., the moments 〈ln(x)〉 and 〈ln(1−x)〉 are conserved during a knowledge compression based on minimal information loss.

#### 2.5.4. Conservation of Reputation

However, one could also argue that the agents rather should care about the reputation of others directly instead of its logarithm, because the former is the quantity the agents actually have to judge during the simulation. For example, in Equation (8) one can see that the expected honesty of the speaker *a* as seen by the receiver *b*, i.e., *b*’s reputation in the eyes of *a*, ${\bar{x}}_{ba}$, plays a crucial role in the cognitive inference performed by the agents. Therefore, another compression method might be more useful, namely one that conserves the reputation itself. Since, however, the beta distribution into which we want the belief state to be compressed is described by two parameters, we can choose another moment to be conserved. For reasons of consistency, since the reputation is the first moment of the probability distribution, we additionally choose the second moment, i.e., the distribution’s variance, to be conserved. In general, we can write down the complex, to be compressed distributions (e.g., Equations (9) and (11)) as $P\left(x|I^{\prime }\right)=y_{J}P\left(x|I^{h}\right)+\left(1-y_{J}\right)P\left(x|I^{\neg h}\right)$ with $I^{h}$ and $I^{\neg h}$ being the new knowledge state given a completely honest or dishonest message, respectively. Then we can calculate the expected honesty and its variance according to

(15) $$\begin{array}{c} \begin{array}{cc} {\bar{x}}_{I^{\prime }} & ={\langle x\rangle }_{\left(x|I^{\prime }\right)}\\ & =y_{J}{\bar{x}}_{I^{h}}+\left(1-y_{J}\right){\bar{x}}_{I^{\neg h}}\\ \sigma_{I^{\prime }}^{2} & ={\langle {\left(x-\langle x\rangle \right)}^{2}\rangle }_{\left(x|I^{\prime }\right)}\\ & ={\langle x^{2}\rangle }_{\left(x|I^{\prime }\right)}-{\bar{x}}_{I^{\prime }}^{2}\\ & =y_{J}{\langle x^{2}\rangle }_{\left(x|I^{h}\right)}+\left(1-y_{J}\right){\langle x^{2}\rangle }_{\left(x|I^{\neg h}\right)}-{\bar{x}}_{I^{\prime }}^{2}\\ & =y_{J}\left({\langle x-{\bar{x}}_{I^{h}}\rangle }_{\left(x|I^{h}\right)}+{\bar{x}}_{I^{h}}^{2}\right)+\left(1-y_{J}\right)\left({\langle x-{\bar{x}}_{I^{\neg h}}\rangle }_{\left(x|I^{\neg h}\right)}+{\bar{x}}_{I^{\neg h}}^{2}\right)-{\bar{x}}_{I^{\prime }}^{2}\\ & =y_{J}\left(\sigma_{I^{h}}^{2}+{\bar{x}}_{I^{h}}^{2}\right)+\left(1-y_{J}\right)\left(\sigma_{I^{\neg h}}^{2}+{\bar{x}}_{I^{\neg h}}^{2}\right){\bar{x}}_{I^{\prime }}^{2}, \end{array} \end{array}$$

where all individual moments are the known moments of a beta distribution:

(16) $$\begin{array}{c} \begin{array}{cc} {\bar{x}}_{I^{h}} & =\frac{\mu^{h}+1}{\mu^{h}+\lambda^{h}+2}\\ {\bar{x}}_{I^{\neg h}} & =\frac{\mu^{\neg h}+1}{\mu^{\neg h}+\lambda^{\neg h}+2}\\ \sigma_{I^{h}}^{2} & =\frac{{\bar{x}}_{I^{h}}\left(1-{\bar{x}}_{I^{h}}\right)}{\mu^{h}+\lambda^{h}+3}\\ \sigma_{I^{\neg h}}^{2} & =\frac{{\bar{x}}_{I^{\neg h}}\left(1-{\bar{x}}_{I^{\neg h}}\right)}{\mu^{\neg h}+\lambda^{\neg h}+3}. \end{array} \end{array}$$

The simplified distribution in the shape of a beta distribution, which the agents will remember can then be constructed using these moments

(17) $$\begin{array}{c} \begin{array}{cc} P(x|I^{\prime \prime }) & =\mathrm{Beta}(x|\mu^{\prime \prime },\lambda^{\prime \prime })\;\;\;\;\;\mathrm{with}\\ \mu^{\prime \prime } & =\frac{-{\bar{x}}_{I^{\prime }}^{3}+{\bar{x}}_{I^{\prime }}^{2}-{\bar{x}}_{I^{\prime }}\sigma_{I^{\prime }}^{2}-\sigma_{I^{\prime }}^{2}}{\sigma_{I^{\prime }}^{2}}\\ \lambda^{\prime \prime } & =\frac{{\bar{x}}_{I^{\prime }}^{3}-2{\bar{x}}_{I^{\prime }}^{2}+{\bar{x}}_{I^{\prime }}\left(1-\sigma_{I^{\prime }}^{2}\right)}{\sigma_{I^{\prime }}^{2}}. \end{array} \end{array}$$

Both approaches are valid compression methods that follow different strategies. While the second one concentrates on the others’ reputations, i.e., on the probability to receive a lie in the next conversation, the first one puts more emphasis on capturing the whole distribution. Thereby the latter gives automatically more weight to the edges of the distribution, i.e., honesties near 1 or 0 due to the logarithms, whereas the other one gives equal weight to all possible honesty values. In Section 3.1, both approaches will be compared in terms of functionality and effects in the reputation game simulations and we will draw a conclusion on which one suits our purposes better.

## 3. Results and Discussion

The results of this work are split into two parts. The first one is about the comparison of the above compression methods, where we discuss the stability of the simulation against that change and conclude which one will be used in the future. The simulations used for the results of the second part then already include the previously chosen compression method. There the focus will be on the analysis of group dynamics as well as the description and understanding of observed emergent phenomena.

### 3.1. Comparison of Compression Methods

In previous works [1,20] we used the optimal belief compression as described in Section 2.5.3, as it follows information theoretical principles and ensures minimal information loss [21]. However, there are two reasons why we now question this choice. On the one hand, this compression method is computationally expensive due to the involved numerical minimization (see Equation (12)). On the other hand, we have seen that this compression method conserves the two moments 〈ln(x)〉 and 〈ln(1−x)〉 and, therefore, puts extreme weight on the very extremes of the distribution, i.e., the agents distinguish much more between honesties of 0.99 and 0.995 than between 0.55 and 0.555 for example. One might argue that these different weights on reputation regimes are actually not useful, and especially not because the logarithms of reputations are not the quantity that later on affects the agents’ decisions, but rather the reputation itself. The second compression mechanism as described in Section 2.5.4, however, could solve both problems. Per the construction, it conserves the reputation of an agent itself including its uncertainty and since the compression can be done analytically (see Equations (15)–(17)) the process would also become much faster. However, we have to ensure that the dynamics are not fundamentally affected by this change, as well as that the agents are still able to judge their environment in terms of reputation equally well (or maybe even better).

To explore the differences we thus consider the three-agent setup used in previous works, which behaves very similarly to the setup discussed in this work. This way we can test the different methods on less expensive simulations and perform the large-scale simulation with 50 agents only once with the selected compression method. The main difference between the two versions is that in the small-scale version, the agents do not track their acquaintances and therefore, the choice of conversation partner and topic does not depend thereon. This is also not needed in the three-agent setup, since the three agents know each other very well and talk to each other in approximately equal parts. For a detailed description of those simulations, please see [1]. As the logical advantage of the second algorithm is the direct conservation of the reputation, which in turn enters in the agents’ credibility judging process (see Equation (8)), we compare the methods by checking how accurate the agents’ judgments are. If there is any difference in the quality of their updates, it should be best visible at this step. To this end, after each credibility judgment, we calculate the surprise that an agent would experience when told whether the message just received was actually honest or a lie. The state space of messages contains only two possibilities, namely the message being honest or not: h∈{h,¬h}, where *h* is considered the random variable. We can then describe the true probability distribution of the message being honest by $\hat{p}\left(h=h\right)=\delta_{hh}$ and $\hat{p}\left(h=\neg h\right)=1-\hat{p}\left(h=h\right)$, with δ being the Kronecker delta symbol. Additionally, we can describe the distribution assigned by the judging agent as

(18) $$\begin{array}{c} \begin{array}{cc} p(h=h) & =y_{J}\\ p(h=\neg h) & =1-y_{J}. \end{array} \end{array}$$

Following information theory, the surprise of an agent with probability distribution *p* if told the true distribution $\hat{p}$ is then given by the Kullback–Leibler divergence

(19) $$\begin{array}{c} \begin{array}{cc} \mathrm{surprise} & =\mathrm{KL}(\hat{p}\left||p\right)=\sum_{X\in \{h,\neg h\}}\hat{p}\left(h=X\right)\ln\left(\frac{\hat{p}\left(h=X\right)}{p(h=X)}\right)\\ & =\delta_{hh}\ln\frac{\delta_{hh}}{y_{J}}+\left(1-\delta_{hh}\right)\ln\frac{1-\delta_{hh}}{1-y_{J}}\\ & =\left\{\begin{array}{cc} -\ln(y_{J}) & \mathrm{if}\;\;\;h=h\\ -\ln(1-y_{J}) & \mathrm{if}\;\;\;h=\neg h \end{array}\right. \end{array} \end{array}$$

**Figure 1:**
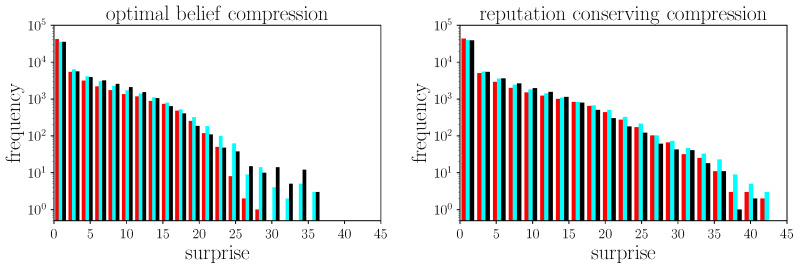
Statistics of the accuracy the agents achieved when judging a received message’s credibility. **Left**: with agents using optimal belief compression. **Right**: with agents using reputation-conserving compression. Specifically, the surprise is measured as what an agent would encounter if it learned the actual honesty status of the message at hand. Different colors indicate the three participating agents, where black stand for the most honest agent, cyan for the intermediately honest agent, and red for the least honest one.

This surprise is calculated in each update during 100 statistical simulations with 300 rounds each, all performed with ordinary agents only. The dynamics of the simulation are not affected by this study, because we do not actually tell the agents the true status of the message, but only calculate and save the surprises in the background. The resulting histograms of occurred surprises can be seen in Figure 1. Here, the three colors indicate the surprises of the three participating agents.

We see that in principle the shapes are similar for both compression methods, but with significant differences in the high-surprise regime. All agents, but especially the least honest one (red), experience fewer high surprises, i.e., judge the credibility of received messages most reliably. Agent cyan and black, in contrast, experience higher surprises, which is due to agent red’s many lies they are confronted with. One can clearly see here that both agent cyan and black are comparatively honest in relation to red, which is why both achieve similar results, but agent red’s differ. In other words, agent red on average has an easier job judging the others than vice versa and in turn, experiences fewer high surprises. The fact that this difference is not visible in the right panel, however, suggests that this effect due to lie distortion is outweighed by the general inaccuracy of the judgments due to the new compression method. All in all, when the agents perform worse with the reputation-conserving compression even in the judgment process, where the speaker’s reputation directly enters, this new compression method should be rejected. Consequently, all the following simulations with 50 agents are based on the optimal compression method.

**Figure 2:**
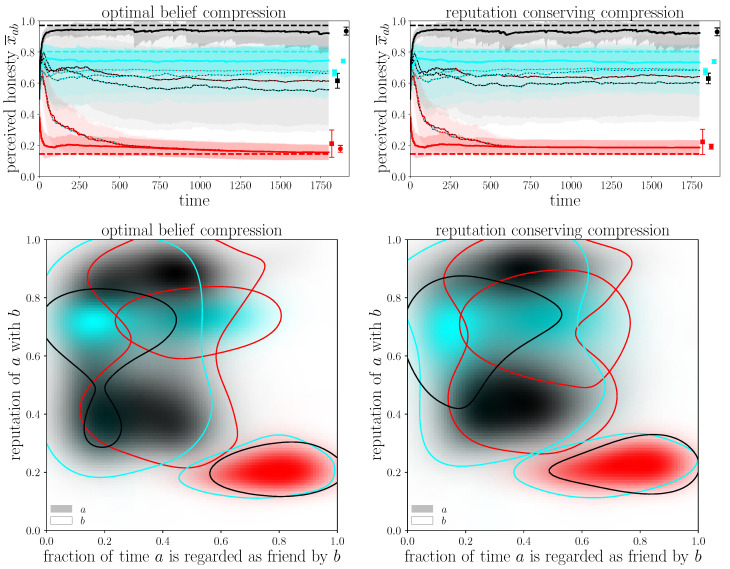
Comparison of statistical simulation results with optimal belief compression (**left**) and reputation conserving compression (**right**). All plots are generated by averaging over 100 realizations of the same simulation with different random seeds. The first row shows the agents’ self esteems (thick lines) and reputations with others (e.g., a black line with red dots indicates agent red’s view of agent black). The dashed lines show the agents’ true, intrinsic honesties and the symbols at the right the agents’ average reputation (square) and self-esteem (circle). The second row shows the correlation between agents’ reputations and the fraction of time they spent as friends. For example, the red region surrounded by a black line in the lower right corner indicates that agent black considers agent red a friend most of the time, while agent black believes agent red to be only around 20% honest.

Nevertheless, we can observe the overall stability of the reputation game simulation against a change in the update mechanism. Therefore, Figure 2 compares the evolution of opinions as well as the correlation between reputation and friendship. In general, we see that the outcome of the simulation is quite similar independent of the used compression method, as left and right plots only differ a little. The main difference is that with the reputation conserving method, all agents overestimate the red agent slightly without an increase in their uncertainty. This might be due to the fact that agents using this compression method misjudge others and especially their opinions slightly, which leads to agent black and cyan being less capable of detecting all lies agent red makes. Thus their opinion of agent red becomes better and in turn, agent red gets better feedback and believes that partially, which is why even agent red’s self-esteem stays higher in the right panel. Additionally, we can see in the lower panels, that both agent red’s and agents black’s opinions on agent cyan’s honesty are not as sharp as in the optimal compression method, indicating that they are less stable in estimating others’ honesties. However, the regime where agent black assigns agent cyan an honesty of only around 40% is less pronounced in the reputation-conserving compression. That means that here the scattering of opinions depending on the random seed is stronger, indicating that this update mechanism is less robust against lies and suggests keeping the optimal belief compression. In general, though, it is worth noting that the simulation results are similar enough to reproduce the characteristic socio-psychological effects, which means that the algorithm is stable against this exchange of knowledge-compression methods.

### 3.2. Analysis of Group Dynamics

For the following analysis, we use a setup with 50 agents with honesties equally distributed from 0 to 1 and 300 conversation rounds. As shown in [22], the agents typically form groups in which they have around 3 to 4 other agents with whom they have had at least 100 conversations. These group sizes are comparable to the system sizes used in [1], where after 100 conversation rounds the simulation was about to reach a steady state. Additionally, we will later see that agents reach quite significant informedness, also suggesting that the presented simulations are sufficiently converged after 300 rounds. Furthermore, Section 2.5.3 describes the optimal belief compression method used, and the personal character traits of shyness and friendship affinity are distributed randomly and differently in each simulation. For statistical significance, we perform 100 realizations of that simulation with different random seeds.

#### 3.2.1. Honesty and Reputation

First of all, we have a look at the fundamental quantity in the reputation game, the agents’ reputation. Here, reputation is the common view others have on a given agent, i.e., the expectation value of an agent’s honesty under the combined belief states of all others. Since there is no coupling between belief states in the reputation game yet (compare Equation (2)), we can define the combined opinion of all others about agent *i* as

(20) $$\begin{array}{c} \begin{array}{cc} P(x_{i}|I_{1i},I_{2i},\ldots ) & =\frac{1}{N}\prod_{j\in A\backslash \{i\}}x^{\mu_{ji}}{\left(1-x\right)}^{\lambda_{ji}}\;\;\;\mathrm{with}\\ N & =B\left(1+\sum_{j\in A\backslash \{i\}}\mu_{ji},1+\sum_{j\in A\backslash \{i\}}\lambda_{ji}\right) \end{array} \end{array}$$

for normalization. With this, we can calculate agent *i*’s overall reputation

(21) $$\begin{array}{c} \begin{array}{cc} {\bar{x}}_{i} & ={\langle x_{i}\rangle }_{\left(x_{i}|I_{1i},I_{2i},\ldots \right)}\\ & =\frac{B(\sum_{j\in A\backslash \{i\}}\mu_{ji}+2,\sum_{j\in A\backslash \{i\}}\lambda_{ji}+1)}{B(\sum_{j\in A\backslash \{i\}}\mu_{ji}+1,\sum_{j\in A\backslash \{i\}}\lambda_{ji}+1)}. \end{array} \end{array}$$

**Figure 3:**
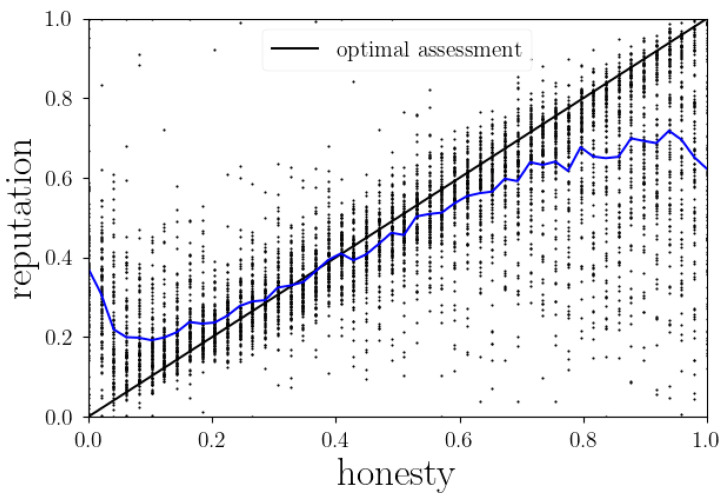
True honesty of agents compared to their reputation by others. Reputation here is a weighted average of others’ opinions by their contact strengths. Black: identity, i.e., how the agents should estimate others’ honesties optimally. Blue: average reputation per honesty.

This definition also ensures that opinions of uninformed agents, i.e., agents that have never (hardly) had any contact with *i*, also do not affect agent *i*’s reputation, because their μ and λ are (near) zero and do not (hardly) contribute to all the sums. In Figure 3 the agents’ reputation is shown in comparison to their real honesties, where perfect reputation assignment would correspond to an identity function. We see that there is a clear tendency for honest agents to be underrated and dishonest agents to be overrated, which in principle was to be expected. Since for a more extreme opinion about someone, much more data is required than to form just a moderate opinion, all belief states naturally tend towards the 50%. Especially in large system sizes where an agent has a high acquaintance typically to just a few others but by far not all who contribute to that agent’s reputation, most of the time the agents simply had not had enough data to sharpen their opinions. This effect, though, can only explain deviations from an extreme towards a neutral opinion and should only cause a flattening at both ends of the average reputation (black curve). However, we observe many simulations where reputation ended up in just the opposite regime as the actual honesty, and at both ends the average curve even changes direction. On the right we can identify the very honest agents being regarded as strong liars as the reputation game realization of the Cassandra Syndrome. This was already found in the three-agent setup of the reputation game simulation (see [1]), and can now also be reproduced in the large-scale version. Furthermore, on the left side of Figure 3 we see a similar, but inverted effect: the strongest liars actually are reputed relatively high, or at least higher than agents with an honesty of around 8%. This is the case because extreme liars, by almost never saying the truth, hardly reveal any information about themselves at all. Mostly the absence of confessions helps the most extreme liars to maintain a completely fake image of themselves. This way, their reputation decouples from their intrinsic honesty, which leads to the increase of reputation at the very low honesty regime. This fact that very dishonest agents only show a fake image and completely hide their true opinions leads to another important effect, which will be discussed in Section 3.2.2.

Besides the effects at the very end of the honesty values, there is another non-trivial observation in the middle of Figure 3. Contrary to expectations, the intersection of the average reputation and the true honesty of an agent is not at 50%. Rather, the border between being overrated and underrated is at an honesty of about 35%, meaning that agents generally tend to judge others less honest than they actually are, i.e., are too critical. This leads to the conclusion that for ordinary agents, the Cassandra syndrome and the associated harm are stronger than the positive effect that lies could have when pretending to be honest. This shows that harming others’ reputations by gossiping is relatively easy compared to increasing one’s own reputation with the help of lies. For now, this is also a relevant feature to keep in mind for the remaining analysis of the reputation game dynamics.

#### 3.2.2. Honesty and Informedness

**Figure 4:**
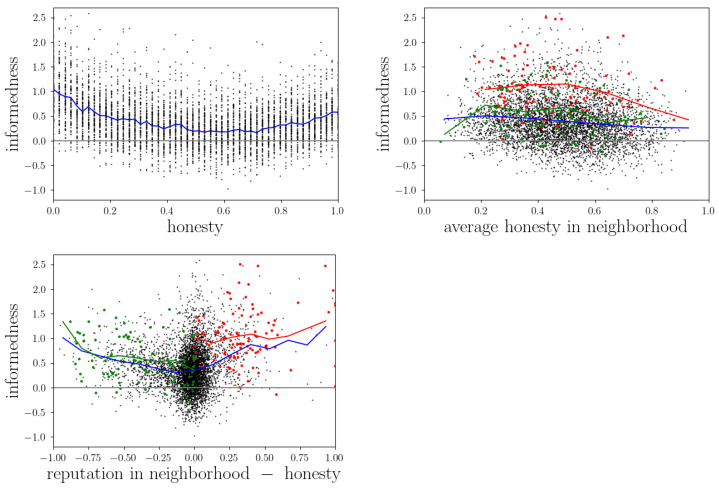
Informedness of agents at the end of simulations and its correlation with their intrinsic honesty (**top left**), the average honesty of their neighborhood (**top right**), and the difference between their reputation and intrinsic honesty (**bottom**). In all plots, the blue line represents the average informedness, and green/red dots and lines the results of the most/least honest agent.

The main goal of the agents is to learn about others’ honesties and to judge them as accurately as possible. Therefore, we introduce an agent’s informedness, which is a measure of how well its worldview is aligned with the ground truth by the end of a simulation. Writing $x_{k}$ as an agent *k*’s true honesty and ${\bar{x}}_{ik}$ as agent *i*’s opinion on *k*’s honesty, we can define agent *i*’s informedness as

(22) $$\begin{array}{c} {\mathrm{informedness}}_{i}=\sum_{k\in A}\left({\bar{x}}_{ik}-\frac{1}{2}\right)\cdot \left(x_{k}-\frac{1}{2}\right), \end{array}$$

which in the 50-agent setup can be any number between $-\frac{50}{12}$ and $\frac{50}{12}$. We can now investigate the correlation of an agent’s informedness with its intrinsic honesty, which is shown in the left panel of Figure 4. Clearly, we see that both very honest and very dishonest agents are better informed than their medium-honest peers. Let us first analyze why dishonest agents are that well-informed. First of all, for any agent to be well-informed, it is important to gain information by talking to others. Since very honest agents per construction have on average to deal with more dishonest agents and vice versa, dishonest agents on average get more reliable information from conversations with others and are less distorted by lies compared to honest agents. This statement can easily be checked by looking at the correlation between an agent’s informedness and the average honesty of their neighborhood. A neighborhood of an agent *i* is hereby defined as the set of all agents that agent *i* has at least talked to once in the simulation. To calculate the average honesty of a neighborhood, we weight the individual honesties of neighbors by the number of conversations they had with the central agent, i.e., by the closeness of the two agents. Thus, the average honesty of a neighborhood N of an agent *i* is given by

(23) $$\begin{array}{c} x_{N_{i}}=\frac{\sum_{n\in N_{i}}r_{in}^{c}x_{n}}{\sum_{n\in N_{i}}r_{in}^{c}}, \end{array}$$

where $r_{ij}^{c}$ is the number of conversations two agents *i* and *j* have had with each other. Following the statement above, one would intuitively assume that the more honest one’s surrounding agents are, the more true information should circulate in this neighborhood and the better should be the informedness of the agents within that neighborhood. However, when looking at the upper-right panel of Figure 4, one does not see a clear correlation for particularly honest or dishonest agents or for average agents. Instead, the best informedness values are reached by agents within a medium-honest environment, but this could as well be a coincidence, because there are only a few very well-informed agents, and the probability of them ending up in a medium-honest environment is higher than in an extreme one. Nevertheless, it is interesting to observe that the honesty of an agent’s neighborhood has no large impact on its informedness, suggesting that the lie detection mechanism of the agents works well enough, such that they are not completely dependent on others’ trustworthiness. On the other side, this could mean that agents are not able to profit from very honest environments, as they probably underestimate the honesty of many of their neighbors in such situations, but we will later see that there is another plausible explanation for this. Anyways, since only an honest environment does not make the agents well-informed, we assume the high informedness of dishonest agents has a more fundamental cause. As already discussed in Section 3.2.1, dishonest agents build up a completely fake image of themselves by hardly revealing any information about themselves at all, which makes it very hard for others to assess their belief states. This in turn would be the basis for others to target their lies towards dishonest agents, because in order to go undetected, agents try to manipulate the receiver’s current estimated opinion when lying (see Equation (7)). However, since other agents can not estimate the dishonest receiver’s current opinion, they can also only badly adapt the lie, which consequentially is more likely to be detected. This way, strong liars maneuver themself in a very good position for detecting lies, which makes them less prone to others’ manipulations and results in higher informedness values. On the other hand, let us now understand why very honest agents are better informed than others. Again, in Section 3.2.1 and Figure 3 we have already seen that honest agents suffer from the Cassandra syndrome quite frequently. This means that often they are not believed although they say the truth, which results in a misjudgment of belief states by their conversation partners. Similar to dishonest agents, this mismatch between honest agents’ belief states and others’ estimations of that leads to easier detectable lies and better informedness.

Let us have a look at this finding from an information trading perspective. Although not implemented in the reputation game yet, information trading is part of humans’ everyday life as one chooses conversation partners, i.e., one’s information sources also consider the expected quality or value of that conversation partner’s statements. To be known as someone whose knowledge is also worth paying for a little bit, e.g., by revealing one’s own valuable knowledge, is not trivial. First of all, one has to be informed in the very first place, which in the reputation game is the case for both very honest and very dishonest agents. Second, however, this must also be recognized by others and even before that, one has to be believed by others. Only if an agent is both well-informed and perceived as honest, i.e., highly reputed it will profit from trading with information. Especially for honest agents, this could be a problem, because they might only be well-informed if regarded as strong liars, as explained before. To prove this argument, the lowest panel of Figure 4 compares an agent’s reputation within its neighborhood with its informedness. Similarly to Equation (21), an agent *i*’s reputation within its neighborhood $N_{i}$ is thus calculated as

(24) $$\begin{array}{c} {\bar{x}}_{i}=\frac{B(\sum_{j\in N_{i}}\mu_{ji}+2,\sum_{j\in N_{i}}\lambda_{ji}+1)}{B(\sum_{j\in N_{i}}\mu_{ji}+1,\sum_{j\in N_{i}}\lambda_{ji}+1)}. \end{array}$$

Moreover, this reputation is then compared to an agent’s real honesty, leading to negative values representing underestimation of an agent’s honesty and positive values representing overestimation. One can clearly see a trend in this panel, where both stronger over- and underestimation lead to better informedness values. This again supports the previous thesis that the misjudgment of other agents is what improved an agent’s informedness. For honest agents (left side), this means that they will often not be able to trade with information, because nobody will recognize the true value of their statements. For liars on the other hand, the informedness level even raises with overestimation, i.e., their trading options seem promising. So if a dishonest agent decides to communicate honest, trustworthy information, it would have a big impact because it is both believed and very informative. If, however, it communicates dishonestly as most of the time, the statement still has a large impact as it is perceived as very valuable, but in reality is just an easy way to misguide the receiver. Actually, this is again a very important insight by itself, because having a large influence with false information makes the judgment of others even worse, which in turn ensures that the dishonest agent will not lose its position, but remain able to detect lies very well, i.e., stay well-informed compared to others.

Besides this effect, the lower panel in Figure 4 also reveals a very simple, but still useful, important insight. Although there are some very extreme over- or underestimations of agents in the left and right edges of the panel, the vast majority of points are located near the center. In particular, most points spread in a range between −0.1 and 0.1, meaning that the agents got their opinions right, mainly with a deviation of less than 10%. This means that for most cases a neighborhood of agents is very well able to assign the right reputation to an embedded agent with reasonably good precision. Thus, we can generally conclude that the modeling approach, and especially the lie-detection and updating mechanisms, i.e., the learning mechanisms implemented in the reputation game, work well on a neighborhood-averaged level.

When again looking at the upper left panel of Figure 4, we obverse a clear asymmetry between dishonest and honest agents. Dishonest ones seem to be even better informed than honest ones. On the one hand this might be due to the fact that dishonest agents always lie very frequently and thereby produce a fake image of themselves, whereas honest agents only suffer from the Cassandra syndrome now and then. However, there might be another self-amplifying effect: as already explained, the others can not judge dishonest agents very well, which makes those good at identifying lies and, therefore, well-informed. However, once a dishonest agent is well-informed, this might in turn help to establish a high reputation, because being well-informed means also being able to target lies (including self-proposing lies) very successfully. This in turn makes others overestimate dishonest agents even more with the result of even worse lies, which brings us back to the origin of the circle. In other words, dishonest agents profit more from being misjudged, because for them, it is in the positive direction, which actually helps to self-amplify this effect and thereby gain even more informedness.

All in all, we observe this interesting effect: albeit intentional or not, hiding one’s own position and at the same time gathering information from others makes the agents well-informed and brings them into a strong position with respect to trading issues. This is also a well-known standard strategy in market business [23] and naturally emerged in the reputation game simulation.

#### 3.2.3. Honesty and Agreement

Another quantity we want to investigate is the agents’ agreement with others and how this is related to honesty. Analogous to informedness, the agreement between two agents *i* and *j* is defined as the alignment of their knowledge vectors:

(25) $$\begin{array}{c} {\mathrm{agreement}}_{ij}=\sum_{k\in A}\left({\bar{x}}_{ik}-\frac{1}{2}\right)\cdot \left({\bar{x}}_{jk}-\frac{1}{2}\right). \end{array}$$

**Figure 5:**
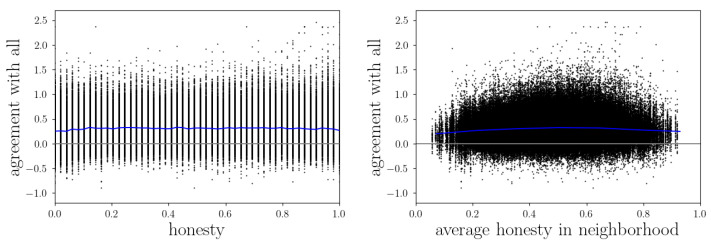
The agreement of agents with all others at the end of simulations with its correlation to their intrinsic honesties (**left**) and the average honesty in their neighborhood (**right**). The color coding is the same as in Figure 4.

In the left panel of Figure 5 we see that on average, an agent’s honesty does not influence its agreement with others very much, but in order to achieve the highest agreement values, an agent has to be very honest. This can be explained because only when an agent gives others the chance to know their true opinions (and they believe them), they can adapt their belief states accordingly. Otherwise, if an agent hides its true worldview, the agreement will never become as high, since others can not adapt as precisely. When comparing both panels, one can conclude that these very honest agents that achieved the highest agreement values have only done so if their environments were quite honest, too. Again there might be too little data to say whether or not the highest agreement values would emerge in the most honest groups because it is just more likely to end up in a moderately honest neighborhood. However, the calculation of the average honesty in an agent’s neighborhood excludes the honesty of that agent itself. Considering that, together with the knowledge that the high agreement values visible in Figure 5 are achieved by very honest agents, we argue that indeed high agreement is only possible if both an agent itself and its environment are honest. The only reason why the peak in the right panel is not at the very right is that there are not many extremely honest agents in the simulation and, therefore, extremely honest neighborhoods with an extremely honest agent in the center are simply very rare. In other words, in extremely honest neighborhoods there has to be a less honest agent in its center, which causes the overall agreement to be lower.

Comparing Figure 5 with Figure 4, the question might arise why for agreements we see a benefit from honest communication, but for informedness we do not. This can be explained by the presence of the illusory truth effect, which we already found to emerge in the reputation game simulations in [22]. It is that the evolving group opinion on which most of the agents agree at the end of a simulation is not necessarily the truth, or at least may deviate from the truth in some aspects. With this in mind, it is clear why agreement and informedness depend on different conditions. For agreement it is only important to find a consensus with others, i.e., revealing all information one has (and believing every received message) would be the optimal strategy, which obviously works best among honest agents. For informedness, however, the crucial point is to distinguish between true and false information, which does not depend on others’ honesties in the first place, but rather on the own ability to judge the environment correctly.

## 4. Conclusions

We saw how in the reputation game simulations reputation networks develop under the core assumption that communication might not always be honest. Both at the individual and group level, we investigated the mechanisms and implications of dishonesty in a large communication system. On the one hand we demonstrated the superiority of one knowledge compression method over another; on the other hand, we measured the quality of the agents’ assessments, including especially their informedness and agreement under certain conditions, and saw a number of socio-psychological phenomena emerge.

First of all, the reputation-conserving knowledge compression method could not outperform the original one and is, therefore, rejected. Even in matters in which it was expected to exceed the original method the simulation showed the opposite. This means that while agents do not conserve each other’s reputation in their knowledge compression, they are ultimately better informed about it than they would be if they conserved it. We note that this suggests that in order to judge someone’s honesty, it is more important to estimate the extremes (near 0 or 1) than to get the middle range correct. This might be a general effect when it is about estimating bounded quantities, because the extreme edges are much more difficult to reach (or occur rarer) than medium values and are, therefore, worth judging with higher precision. This effect might be especially visible in the reputation game simulation due to the central role that honesty estimates play here: on the one hand, the full range of honesties is exhausted and on the other hand, its correct assessment is crucial for staying oriented in such a lie distorted system.

Moreover, we have shown that the reputation game simulation in its large-scale setup is able to capture some interesting real-life phenomena. On the one hand, we have reproduced the Cassandra syndrome, which already emerged in the small-scale reputation game simulations. Additionally, we saw how the construction and maintenance of fake images of oneself helped to significantly increase one’s reputation. Such hiding (albeit intentionally or not) of opinions was also shown to make the agents clearly better informed and thereby stronger in potential trading issues, which again naturally emerged in our simulations and represents a fundamental strategy in the real-life market business. Last but not least, we demonstrated the fundamental difference between informedness and agreement. In every system that contains dishonest communication and suffers from the illusory truth effect, being in good agreement with others and being well-informed were shown to be based on two separate mechanisms. While the former relies on two honest communication partners that trust each other, the latter mainly depends on the capability of an agent to judge the trustworthiness of its environment correctly. Consequentially, agreement and informedness may develop independently of each other, and thus should not be confused. Additionally, for future development of the reputation game simulation and other similar models, this should be kept in mind.

More generally, by reproducing these effects, the large scale version of the reputation game simulation has proven to be a useful tool in the research of emerging socio-psychological phenomena. To follow up in this direction we think of several improvements. First of all, in order to counteract the natural occurrence of many more average-honest neighborhoods than extreme ones, we could think of predefining environments, e.g., by specifying the network graph already at the beginning of a simulation. This way we could gain more statistical weight on extremes, which makes the evaluation of emerged phenomena more expressive. However, we would lose the opportunity for the agents to choose their preferred conversation partners, which could be particularly severe when looking at the next idea: we also consider it necessary to tune the agents’ orientation within the network, i.e., providing them with better guidance. For example, to simulate the natural urge of people for interesting, exciting, or valuable information, we would need another observable that the agents keep track of. On top of honesty and friendship we thus could introduce some measure of competence that the agents steer for. For this to work, though, the network must not be fixed or at least partially adaptable. Only this way can the agents choose their conversation partners depending on their personal needs and interests similar, to how humans constantly choose and change their favorite information sources.

## Data Availability

Data available at https://doi.org/10.5281/zenodo.7387341.
